# Supplementary material for: Impact of COVID-19 on out-of-hospital cardiac arrest: A registry-based cohort-study from the German Resuscitation Registry
Source: PLoS One. 2022 Sep 14;17(9):e0274314. doi: 10.1371/journal.pone.0274314 (PMC9473624; doi:10.1371/journal.pone.0274314)
Supplement: Attachment — Submitted filename: Response to Reviewers.docx [file pone.0274314.s003.docx]

June 11, 2022

Dear Dr. Ballotta,

dear reviewer,

Thank you for the time and effort you have put into reviewing our manuscript entitled “Impact of COVID-19 on out-of-hospital cardiac arrest: a registry-based cohort-study from the German Resuscitation Registry”.

We would like to respond to your comments in detail below. You can see all the changes in the attached version with the track changes.

Comment #1: At line 47 is not fully clear the proportion of the OHCA

Answer #1: Thank you for the comment. We have worded it more clearly.

Comment #2: It might be interesting know the number of COVID positive patients in the cohorts to understand where COVID is implied in the incidence of the event

Answer #2: To our regret, the COVID status of patients is not surveyed by the German Resuscitation Register. Therefore, we are unfortunately unable to make any statements about this.

Comment #3: Is the high quality number of cases influenced by the pandemic period or the proportion is the same?

Answer #3: In the pandemic period, the share of cases from the high quality data group in the total cases was lower than in the non-pandemic period. We took this into account by comparing only those sites that belonged to the high quality data group in both periods.

Furthermore, where necessary, we have adapted formatting according to the specifications of PLOS One.

Our COIs presented in our COI forms do not alter our adherence to  PLOS ONE policies on sharing data and materials. We welcome PLOS One's policy of publishing a minimal data set with the article. You will find this attached for publication (S1 table and S2 table).

Please feel free to contact me at any time if you have any queries concerning our manuscript.

Thank you for your consideration. I look forward to hearing from you.

Sincerely,

Stephan Seewald, MD

On behalf of all authors.

University Hospital Schleswig-Holstein

Institute for Emergency Medicine *and* Department of Anesthesiology and Intensive Care Medicine

Arnold-Heller-Straße 3

24105 Kiel, Germany

Phone	+49 431 500-31551

Fax	+49 431 500-31504

E-mail	[stephan.seewald@uksh.de](mailto:stephan.seewald@uksh.de)
